# Trans fatty acid intake among Chinese population: a longitudinal study from 1991 to 2011

**DOI:** 10.1186/s12944-020-01247-1

**Published:** 2020-04-27

**Authors:** Liying Jiang, Junjie Shen, Yuxia Zhao, Jianwen Li, Sana Liu, Yujie Liu, Huijun Wang, Chang Su, Xun Zhuang, Nianhong Chen, Aidong Liu

**Affiliations:** 1 Shanghai Key Laboratory for Molecular Imaging, Shanghai University of Medicine & Health Sciences, Shanghai, P. R. China; 2 Zhoupu Hospital in Pudong New Area, Shanghai University of Medicine & Health Sciences, Shanghai, P. R. China; 3 Department of Epidemiology, School of Public Health, Nantong University, Nantong, Jiangsu Province, Nantong, 226010 P. R. China; 4 Department of Nutrition Division III, China National Center for Food Safety Risk Assessment, Beijing, 100022 P. R. China; 5 Department Of Public Nutrition And Nutrition Policy, Chinese Center for Disease Control and Prevention, Beijing, P. R. China; 6 Laboratory of Signal Transduction, Department of Radiation Oncology, Memorial Sloan-Kettering Cancer Center, 1275 York Avenue, New York, NY 10065 USA

**Keywords:** Trans fatty acids, Longitudinal study, Food sources, General population

## Abstract

**Objective:**

This study was aimed to roughly describe individual Trans Fatty Acids (TFAs) intake and the percentage of energy intake(*E*%), and identify major food sources in the Chinese population, taking gender, age, and regional distribution into the consideration, as well as examining temporal changes over the course of 20 years.

**Method:**

This multi-center study, covering nine provinces among populations aged ≥ 3 in China, was conducted to collect food consumption information from 1991 to 2011. A classical assessment method was used to estimate the level of dietary TFA intake.

**Results:**

Over the 20-year period, the intake of TFAs in Chinese populations had increased, but remained at a relatively lower level (from 0.25 g/d(0.11% for *E*%) to 0.53 g/d(0.24% for *E*%)) compared with that of other countries and the World Health Organization (WHO) recommended level. Collectively, males and participants aged 19–60 generally consumed more TFA-containing foods. People in eastern regions consumed more TFAs and had a higher *E*% than those in western area. Industrial sources of TFAs, especially vegetable oil, ranked as the principal food sources of TFAs in the Chinese population. Natural sources of TFAs have gradually increased in proportion among children and adolescents.

**Conclusions:**

TFA intake and the *E*% are commonly under the recommended level in the general population in China. Presently, restriction of vegetable oil could be a crucial method to reduce TFA intake. It would be critical to facilitate and promote public health that food recommendations might be based on the dietary preferences for population separated by different ages and regions.

## Introduction

Trans fatty acids(TFAs) refer to unsaturated fats with at least one non-conjugated carbon-carbon double bond in the trans configuration and exist extensively in many foods, from natural origins and industrially processed sources [1]. Dietary TFAs can be of natural or artificial origin, including: (i) milk, milk products, and meat from ruminant animals; and (ii) fat processing that occurs via partial catalytic hydrogenation of oils and fats, commercial refinement of polyunsaturated fatty acids, and oil cooking at very high temperatures [2].

The implications of TFAs for public health have received overwhelming attention. The adverse effects of dietary TFA intake on coronary heart disease (CHD) are well established [3–7]. It has been estimated that 72,000 cardiovascular disease-related deaths per year could be averted by a 1% reduction in the intake of industrially produced TFAs [8]. Furthermore, high TFA intake is generally considered as a risk factor for weight gain [9], although the evidence for the relationship between TFA intake and diabetes is inconsistent [10–14]. A recent meta-analysis confirmed that TFA intake is associated with all-cause mortality and coronary heart disease mortality [15].

Diverse levels of TFAs have been detected worldwide because of different dietary habits and varying quantities of industrial TFAs added to processed foods [16]. Different approaches have been implemented to reduce the amounts of TFAs in processed foods. Indeed, it has been recommended that TFA limits should be established, because they represent the optimal solution when considering both CVD prevention and associated-cost savings in public healthcare [15]. To date, most European countries have established regulations that limits the TFA content in foods and many measures have been adopted to reduce the general population’s intake of TFAs [17–19]. In the United States, multiple strategies have been invoked to limit dietary TFAs, including listing TFAs on the nutrition facts panel of foods and legislation banning fats and/or oils containing TFAs [20]. Meanwhile, dietary recommendations stating that the total energy intake from TFAs should be less than 1% for adults per day (WHO) [21].

In China, previous studies assessing TFA intake could not be representative because of the restricted list of food items and the relatively small samples [22, 23]. A recent study, conducted in Beijing and Guangzhou, was insufficient to accurately and comprehensively reflect the TFA intake of the general Chinese population [24]. Presently, TFA consumption data are unavailable to examine food sources, amounts of TFAs consumed, or temporal changes using national dietary surveys in China. The dietary habits and lifestyle among Chinese people have changed markedly during the last few decades. There is an urgent need to find out TFAs intake in the Chinese population and further provide a better understanding of major food sources of TFAs.

Therefore, our study were conducted to assess TFA intake and energy-percent among people aged ≥ 3 years, in which it was taking gender, age and regional distribution into consideration, and exploring temporal changes in TFA intake from various food origins via a 20-year longitudinal study. These findings will help to provide the evidence-based dietary advice and identify a benchmark for TFA intake, and will assist in the development and monitoring of nutrition and food policies and public health messages.

## Materials and methods

### Study design and participants

Data, deriving from a collaborative project, the China Health and Nutrition Survey (CHNS), were collected from 1991 to 2011. CHNS is a multicenter profile covering nine provinces (Northeast China: Heilongjiang, Liaoning; East Coast: Shandong, Jiangsu; Central China: Henan, Hubei, Hunan; and Southwest: Guizhou and Guangxi), and is an ongoing longitudinal study established in 1991 by the Chinese Center for Food Safety Risk Assessment (CFSA) and the Chinese Center for Disease Control and Prevention (CDC). A multistage, random cluster process was employed to draw a sample in each province. In each province, a weighted sampling scheme was employed to randomly select four counties and two cities. Villages and townships within the counties, as well as urban and suburban neighborhoods within the cities, were selected randomly. In each community, 20 households were selected randomly and all members underwent an interview. Details of CHNS had been described elsewhere and the response rate was about 90% [25].

Subjects aged 3 years and over, with complete data for their socio-demographic characteristics, anthropometric measures, and dietary surveys, were selected to participate in the present study. We excluded participants with missing data for the critical variables. Two hundred sixteen sites (communities), including 36 urban neighborhoods, 36 suburban neighborhoods, 36 towns and 108 villages, were recruited into the study. Finally, a total of 103,326 participants were involved in the analysis. More than 10,000 participants were analyzed in each survey year. All participants provided written informed consent. The study protocol was reviewed and approved by the Ethics Committee of the China Center for Disease Control and Prevention.

### Dietary data collection

In the present study, we collected daily consumption data from all household members using the same three nonconsecutive 24-h food recalls by showing food tables and picture aids, including food categories, amounts, and cooking methods. Household members were prompted to provide information to calculate the amounts of food items consumed at home. The amount of each dish was estimated from the household inventory and the proportions of each dish consumed were reported by each individual. Considering daily variations in food intake, participants were interviewed on two consecutive weekdays and one weekend day.

For children younger than 12, the mother, or whoever who handled food preparation and feeding in the household, was asked to recall children’s food consumption. The survey was conducted by trained staff using a uniform questionnaire. The anthropometric measurements were taken at community health service centers.

### Assessment of the TFA content

The TFA content of each food item was assessed using a traditional analytical method (capillary gas chromatography). The detailed methods have been described previously [24]. In this study, 2613 prevalent food items were systematically collected, 654 of which were found to contain TFAs and were divided into 15 categories according to China Food Composition (2nd Edition). These foods were considered as the main contributors to the TFA intake of Chinese population.

### Estimation of TFA intake and the percentage of total energy intake

Based on the amount of each ingredient in each food consumed during the survey period, we estimated the TFA intake from the reported food. The dietary TFAs intake for each individual was estimated by summing TFA contribution of each food item (the corresponding TFA content), and expressed as daily intake (g/day). Furthermore, we performed analyses concerning TFA intake as the percentage of total energy intake (*E*%). *E*% was calculated as follows: *E% = DI×* 9/*DE×* 100%*.*

Where *DI* was the individual TFA intake per day (g/day) and *DE* was total dietary energy intake for the individual(kcal), which was converted from the individual’s intake of all foods, according to the food energy conversion table in China Food Composition (2nd Edition) on the basis of the individual dietary intake data during the survey period. The energy transfer index of TFA was 9 kcal/g [26].

### Statistical analyses

Body mass index (BMI) was calculated by dividing the weight (kg) by the squared height (m^2^), and the participants were divided into four categories according to the Working Group on Obesity in China recommended criteria(Underweight: BMI < 18.5 kg/m^2^; Normal: 18.5 ≤ BMI < 24; Overweight: 24 ≤ BMI < 28; and General obesity: BMI ≥ 28)[27]. We applied one-way analysis of variance (ANOVA) for continuous variables and a post hoc multiple comparisons were performed using least significant difference-t (LSD-t) tests. Simple linear regression was carried out to analyze the association between individual TFAs intake and survey year, and the applicable conditions of the model were tested. For all tests, P < 0.05 was considered as statistically significant. All statistical analyses were conducted using Stata version 13.0(StataCorp, College Station, TX, USA).

## Results

### Chinese individual TFAs intake with temporal changes

The basic characteristics of the participants in terms of gender, age and BMI are shown in Table 1. The average BMI presented an increasing trend from 20.2 to 23.2 in the longitudinal study. Table 2 shows the energy and TFAs intake separated by age, gender and food source. Chronologically, energy intake for participants had increased at first and then declined, while their intake of TFAs had increased steadily from 0.254 g/d (0.110% for *E*%) to 0.530 g/d (0.235% for *E*%). The simple linear model suggested there was a strong association between TFAs intake and survey time (*β =* 0.013, *P* < 0.001). Similarly, a strong association between *E*% and survey time was also detected (*β =* 0.006, *P* < 0.001).

**Table 1:** Basic characteristics of participants in the study from 1991 to 2011

	1991	1993	1997	2000	2004	2006	2009	2011
**Number of participants (%)**								
Women	6247 (51.9)	5837 (51.6)	6124 (50.4)	6030 (51.0)	5894 (51.3)	5697 (51.4)	5830 (51.0)	5623 (52.1)
Men	5790 (48.1)	5470 (48.4)	6041 (49.6)	5799 (49.0)	5600 (48.7)	5378 (48.6)	5594 (49.0)	7092 (47.8)
Total	12,037	11,307	12,168	11,829	11,494	11,075	11,424	10,790
**Age group (years)(%)**								
**Mean ± SD**	31.4 (0.2)	32.6 (0.2)	35.5 (0.2)	37.8 (0.2)	41.9 (0.2)	43.7 (0.2)	44.7 (0.2)	46.1 (0.2)
≤ 18	3912 (32.5)	3509 (31.0)	3165 (26.0)	2732 (23.1)	2043 (17.8)	1718 (15.5)	1620 (14.2)	1487 (13.8)
19–60	6920 (57.5)	6580 (58.2)	7421 (61.0)	7377 (62.4)	7361 (64.0)	7084 (64.0)	7223 (63.2)	6548 (60.7)
≥ 61	1205 (10.0)	1218 (10.8)	1582 (13.0)	1720 (14.5)	2090 (18.2)	2273 (20.5)	2581 (22.6)	2755 (25.5)
**BMI (kg/m**^**2**^**) (%)**^**a**^								
**Mean ± SD**	20.2 (0.1)	20.4 (0.1)	21.0 (0.1)	21.6 (0.1)	22.2 (0.1)	22.4 (0.1)	22.5 (0.1)	23.2 (0.1)
< 18.5	3654 (31.3)	3224 (29.3)	2666 (24.7)	2374 (20.6)	1772 (16.6)	1543 (15.1)	1636 (15.3)	2031 (14.1)
[18.5,24)	6457 (55.3)	6148 (55.9)	5916 (54.9)	6139 (53.3)	5649 (52.8)	5366 (52.5)	5330 (49.8)	6741 (46.6)
[24,28)	1308 (11.2)	1388 (12.6)	1767 (16.4)	2371 (20.6)	2540 (23.7)	2562 (25.1)	2841 (26.6)	4090 (28.4)
≥ 28	260 (2.2)	246 (2.2)	424 (3.9)	639 (5.5)	734 (6.9)	748 (7.3)	892 (8.3)	1560 (10.8)

^a^The number of people with effective BMI was lower than the total number of people surveyed

**Table 2:** Energy and TFA intake of participants in the study from 1991 to 2011

Years	1991	1993	1997	2000	2004	2006	2009	2011
**Energy intake (kcal/d)**								
**Mean ± SD**	2197 (7.4)	2147 (7.3)	2324 (7.5)	2240 (6.9)	2232 (8.0)	2249 (7.9)	2103 (6.8)	2021 (7.4)
Women	2103 (9.3)	2050 (9.1)	2158 (9.4)	2077 (8.6)	2064 (10.0)	2071 (9.8)	1927 (8.4)	1856 (9.1)
Men	2299 (11.4)	2251 (11.2)	2493 (11.4)	2409 (10.4)	2409 (12.1)	2437 (12.1)	2286 (10.3)	2201 (11.4)
≤18	1807 (11.6)	1769 (11.6)	1916 (13.8)	1873 (13.5)	1814 (17.5)	1748 (17.8)	1628 (16.8)	1557 (17.8)
19–60	2431 (9.3)	2359 (9.2)	2548 (9.2)	2413 (8.3)	2381 (9.6)	2417 (9.7)	2244 (8.2)	2166 (9.3)
≥61	2126 (20.7)	2098 (20.0)	2092 (18.1)	2080 (17.0)	2116 (18.7)	2102 (16.5)	2005 (13.6)	1928 (13.9)
**Total TFA intake(g/d)(*E*%)**^**a**^								
**Mean**	0.254 (0.110)	0.267 (0.116)	0.367 (0.143)	0.387 (0.155)	0.430 (0.170)	0.480 (0.193)	0.471 (0.202)	0.530 (0.235)
Women	0.243 (0.109)	0.250 (0.115)	0.246 (0.144)	0.364 (0.157)	0.408 (0.174)	0.447 (0.195)	0.437 (0.204)	0.499 (0.240)
Men	0.266 (0.111)	0.284 (0.118)	0.389 (0.142)	0.412 (0.153)	0.453 (0.166)	0.515 (0.191)	0.506 (0.199)	0.563 (0.230)
≤18	0.197 (0.104)	0.201 (0.108)	0.299 (0.142)	0.320 (0.153)	0.379 (0.181)	0.380 (0.194)	0.407 (0.223)	0.429 (0.246)
19–60	0.284 (0.111)	0.297 (0.119)	0.399 (0.142)	0.410 (0.152)	0.449 (0.167)	0.511 (0.191)	0.496 (0.198)	0.562 (0.234)
≥61	0.269 (0.120)	0.293 (0.127)	0.354 (0.149)	0.398 (0.170)	0.415 (0.173)	0.459 (0.197)	0.441 (0.199)	0.506 (0.232)
Natural source	0.045 (0.020)	0.056 (0.026)	0.062 (0.025)	0.075 (0.031)	0.095 (0.040)	0.094 (0.040)	0.096 (0.043)	0.094 (0.044)
Industrial source	0.209 (0.090)	0.210 (0.091)	0.305 (0.118)	0.312 (0.124)	0.335 (0.131)	0.386 (0.153)	0.375 (0.159)	0.435 (0.191)

^a^The standard deviations are all between 0.002 and 0.006

**Fig. 1:**
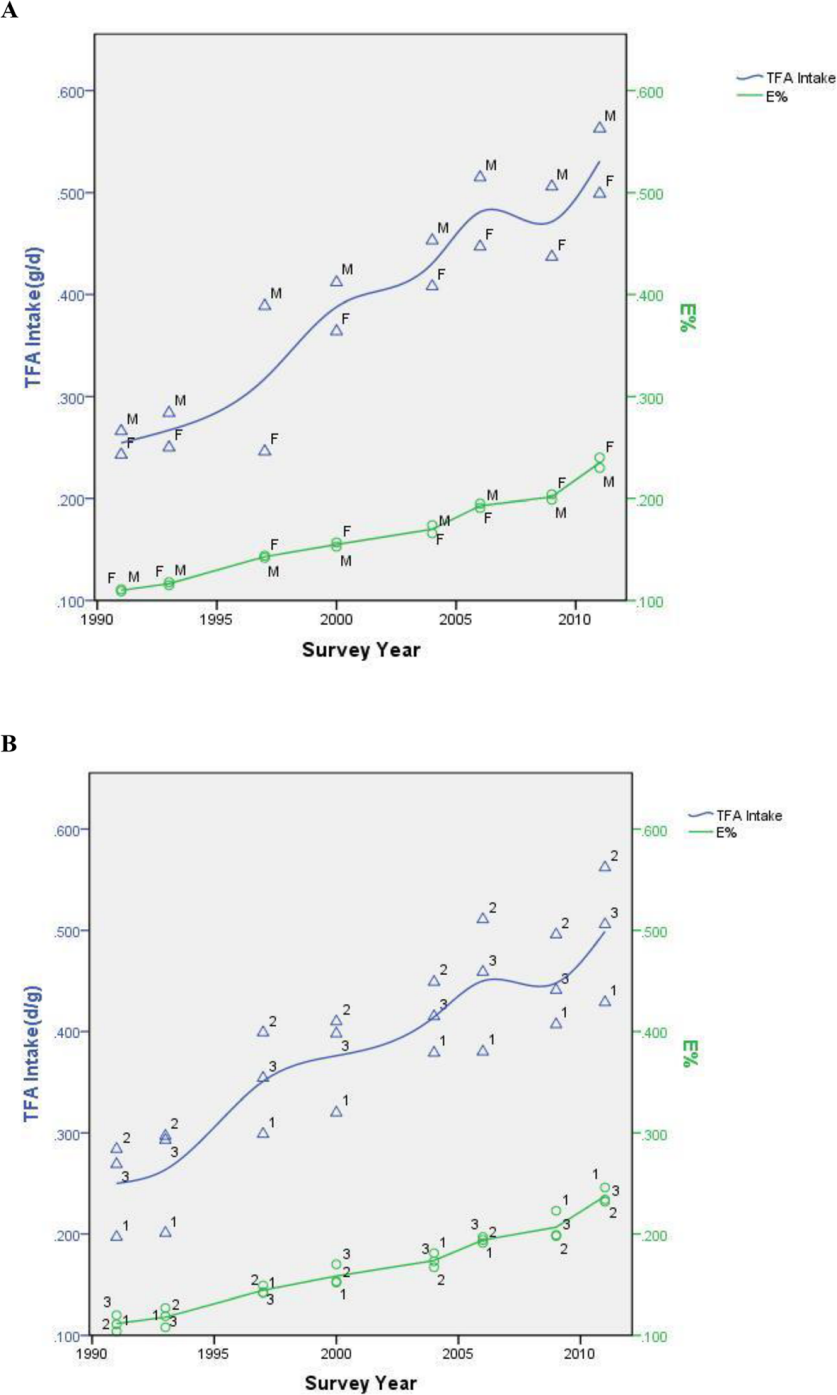
**a**. TFA intake and *E*% by sex in each survey year. **b**. TFA intake and *E*% by age groups in each survey year

Figure 1 shows the variation tendency of TFA intake and *E*% separated by gender and age over the years. Generally, males took in more TFAs than females. People aged 19–60 had the highest level of TFA intake and children and adolescents (≤18) commonly took in the least TFAs. However, in terms of *E*%, there was no significant difference between genders (Supplementary Table1). *E*% for group aged ≤ 18 presented a higher value than that of adults and older age population in the recent four survey years (Supplementary Table 2).

### TFAs intake across China

**Fig. 2:**
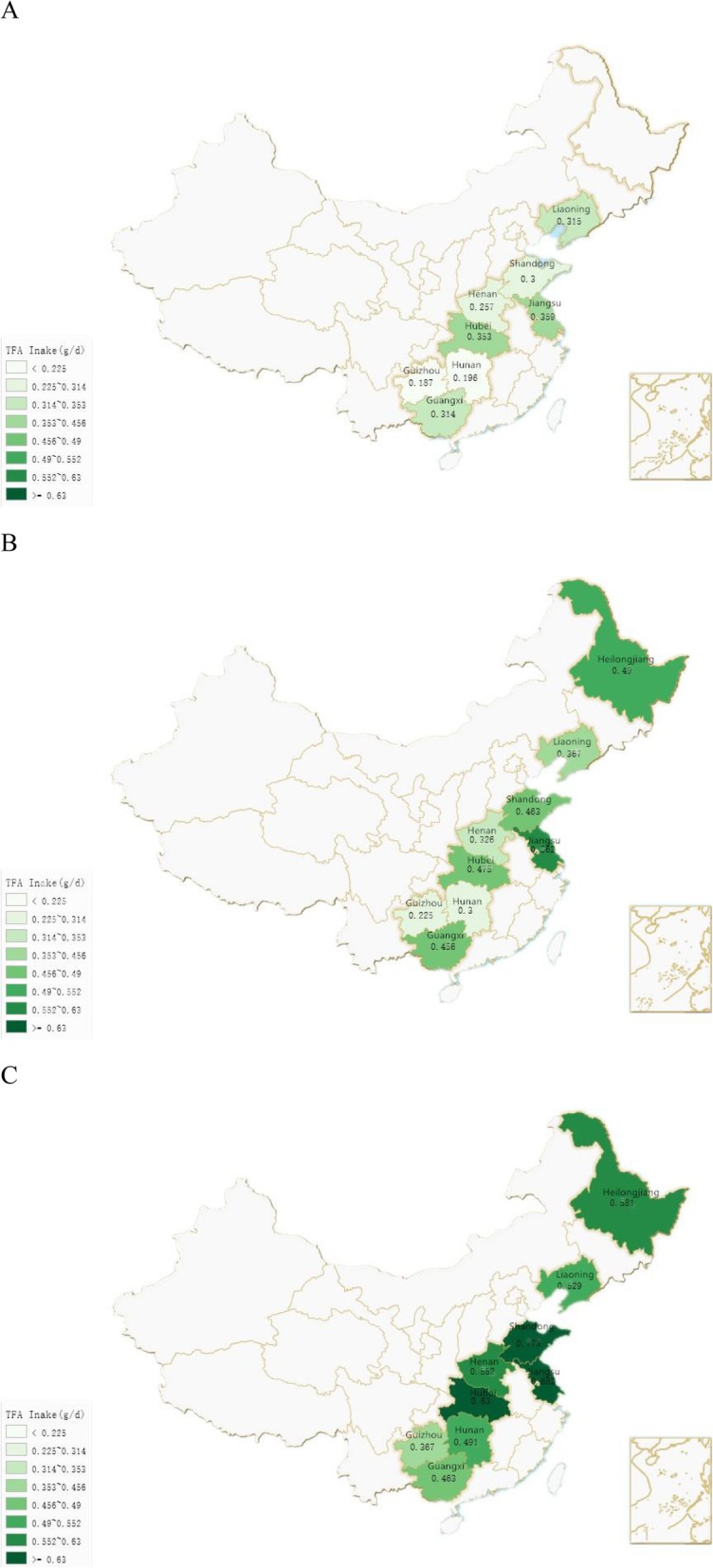
**a**. TFA intake of 19–60 years group in each survey province in year 1991. **b**. TFA intake of 19–60 years group in each survey province in year 2000. **c**. TFA intake of 19–60 years group in each survey province in year 2011

Supplementary Table 3 shows the TFA intake (g/d) of participants separated by ages in different provinces in survey years. As noted, participants aged 19–60 generally consumed more TFAs. Further comparable analysis stratified by different regions and ages in survey years was conducted (Supplementary Table 4). TFA intake in absolute amounts in the middle and southern regions, especially Guizhou and Hunan (except Hubei), had kept a much lower value when compared with that in the northern and eastern regions in the longitudinal years. Figure 2 shows TFA intake of 19–60 years groups in the survey provinces in the year 1991, 2000 and 2011.

**Fig. 3:**
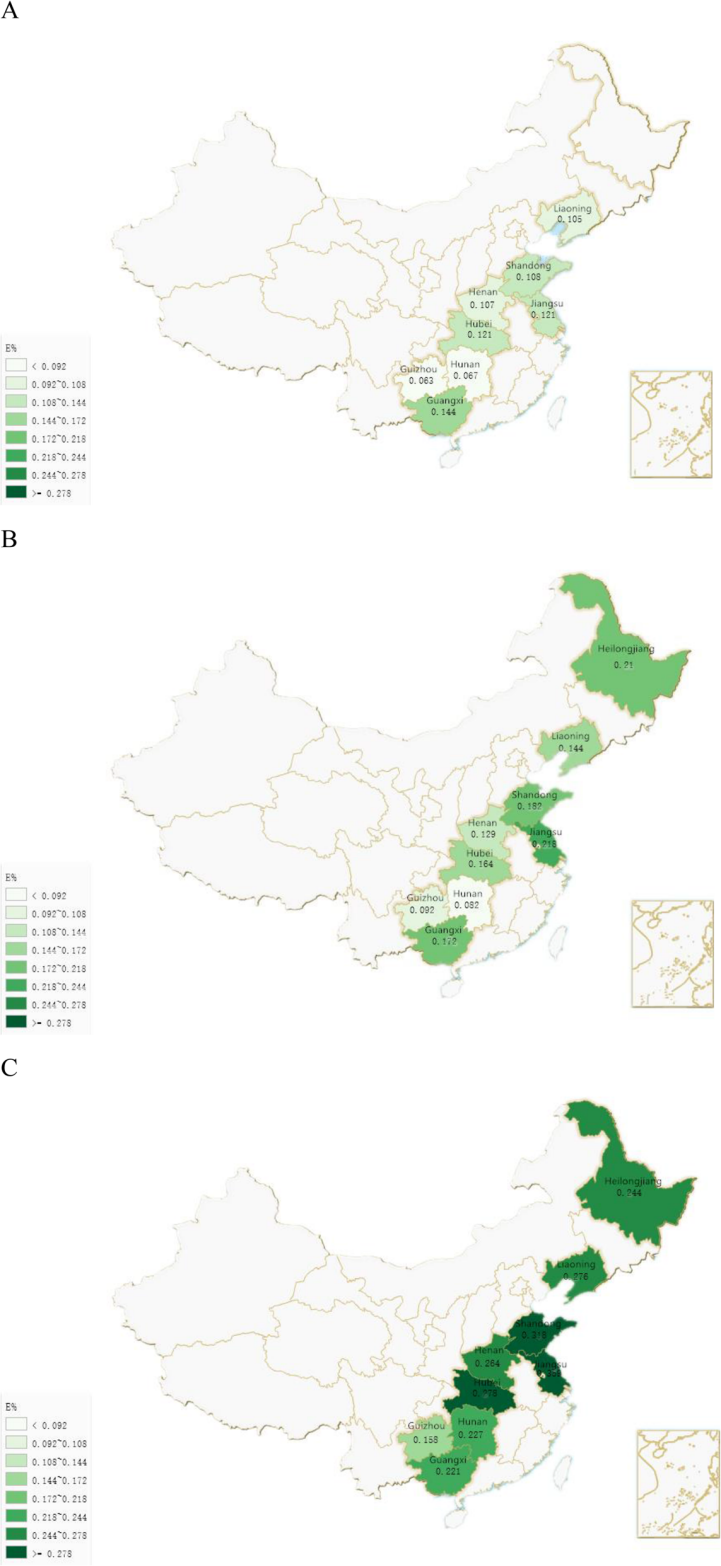
**a**. *E*% of the children and adolescents group in each survey province in year 1991. **b**. *E*% of the children and adolescents group in each survey province in year 2000. **c**. *E*% of the children and adolescents group in each survey province in year 2011

Supplementary Table 5 shows the details for TFA intake as *E*% by ages in these provinces in survey years. Further analysis shows that population in Guizhou and Hunan had presented a relatively low level of *E*% (0.061–0.200%), while Jiangsu and Shandong had reached much higher since 2000. The rest provinces presented minor difference (from 0.102 to 0.278%). Figure 3 shows *E*% of group aged ≤ 18 in these provinces in the year 1991, 2000 and 2011(Supplementary Table 6).

### Food source of Total TFAs

Supplementary Table 7 presents the profile of foods’ contribution to total TFAs over the 20-year period by age. Collectively, vegetable oil ranked as the principal industrial sources of TFAs for all the population in the longitudinal time, although its contribution to TFAs had gradually decreased as time went on. As one of the key natural TFA intakes, the contribution from milk and milk products was steadily arising. As another critical industrial source of dietary TFAs, bakery products presented an increasing trend for all the population.

For participants under 19 years, the proportion of natural sources of TFAs had gradually increased among children from 18 to 30%, while that of industrial sources of TFAs had correspondingly declined. Interestingly, the contributions from the natural and industrial sources were relatively stable (natural sources: 16–20%) in participants aged 19–60 and ≥ 61. Notably, the contribution of fried foods, ethnic foods, bakery products, fast foods, youbing and youtiao, all presented a stable rising trend. Furthermore, butter and chocolate contributed less than 1% of TFAs intake because of their lower consumption, although TFA levels in food origins were much higher.

## Discussion

TFA intake has been identified as a potential health hazard in both adults and preschool children. In order to improve our knowledge, this study described the TFA intake and the *E*% in a general population with reasonable coverage across the whole country. Our study showed that the mean TFA intake of study population increased from 0.25 g/d to 0.53 g/d, more than double times during the last 20 years. It also suggested that younger and middle-aged people, as well as males, generally consumed more TFAs in the daily life. For its origins, industrial TFAs constituted a relatively large portion, taking about 80% of the total TFAs intake. The average intake of TFAs varied considerably because of variations of dietary patterns over the last two decades. The proportion of foods containing TFAs had been continuously increasing, and these foods primarily comprised dairy products, meats products, and fried foods.

When expressed as *E*% in all populations across different years, an increasing trend was observed (0.11 to 0.24%). Collectively, the difference in *E*% between male and female was non-significant among different ages. The *E*% of the children and adolescents tended to increase faster than that of other two age groups over the last 5 years. This trend was consistent with the findings that adolescents were exposed to a higher proportion of TFAs [20, 24]. Additionally, the abundant consumption of dairy products had become an important source of dietary TFAs.

In terms of geographical distribution, the *E*% increased from the west regions to the east regions, the *E*% value for participants in Jiangsu and Shandong were similar to the study in 2011 [24]. Nationwide, the highest level in 2011 was 0.36%, which was still far under the dietary’s recommendations of WHO(1%) and United States during 1999 to 2002(2.5%) [20]. Meanwhile, comparing with Japan’s *E*% with 0.80% in 2010 [28] (Japan is probably one of the countries consuming the least TFAs) the *E*% from TFAs in China was still lower. Chinese traditional dietary patterns, higher consumption of natural food sources (cereals and vegetables, etc.) with no TFAs and fewer industrial TFA-containing foods, which might contribute to this relatively lower value. Edible vegetable oil, meat products, milk and milk products, being necessary in daily meals, accounted for more than 80% of the TFAs in the study population. As noted, the major food sources of TFAs were bakery foods, such as cakes, cookies, pies, pastries and confectionary in USA [20, 28]. In our present study, vegetable oil ranked as the top food source of TFAs, although its contribution had gradually decreased as time went on. Most importantly, it is critical to restrict the consumption from each item of foods, rather than the TFAs content of foods.

Several characteristics of the study should be noted. Firstly, there was no study until now, but our study described the TFAs intake in the general Chinese population and comprehensive view of food sources were based on contextual data because of its long duration and wide geographical coverage. Secondly, three 24-h recalls within 3 days of intake data were considered to increase the validity and precision compared with the individual 24-h only recall method. Moreover, as a simple assessment of food intake, its evaluation for TFAs from food origins was sufficiently accurate. In addition, 2613 categories were considerably large enough to cover TFA-containing foods for the Chinese foods inventory.

There were still some potential limitations that should be considered. Firstly, the level of plasma TFAs were unavailable, which could help to illustrate the metabolic processes and deleterious effects on health. Notably, there was a well-established correlation between the plasma concentration of TFAs and the TFAs intake [29]. Secondly, we employed food TFA levels measured in recent years (2011) to simulate food TFA levels over the past 20 years (1991–2011), which was likely inaccurate. Over time, the process of processed food had changed the TFAs content. However, our research suggested that the majority of dietary TFAs were mainly derived from plant oils and natural foods, with little TFAs from processed foods. Additionally, TFAs content in natural foods and that produced in vegetable oil processing were relatively stable, which could roughly describe their past TFAs intake. Thirdly, the hazardous effects of different TFAs subtypes on key outcomes, such as all-cause, CVD, and cancer mortality could be unavailable. Estimation of TFAs consumption was appropriate for total TFAs but not subtypes of TFAs (saturated fatty acids, SFAs; monounsaturated fatty acids, MUFAs; and polyunsaturated fatty, PUFAs). Therefore, we could only describe total TFAs consumption captured by dietary estimations. Despite of that, our study still could serve as a popular proxy for the consumption of TFAs.

## Conclusion

In summary, TFA intake and the percentage of energy intake were commonly under the recommended level in the general population in China. At present, restriction of TFAs from vegetable oil could be a crucial procedure. It would be critical to facilitate and promote the health that food recommendations might be based on the dietary preferences for population separated by different ages and regions. Concerted efforts, including production technology improvements, nutritional labeling reinforcement, and public health education, should be encouraged to further reduce the amount of TFAs intake in the dietary pattern in China.

## Supplementary information

**Additional file 1 : Supplementary Table 1.** T test of TFA intake as the percentage of total energy intake by age-specific distribution in specific gender in each survey year (%). **Supplementary Table 2.** Post hoc test of TFA intake as the percentage of total energy intake in age-specific distribution by gender in each survey year (%). **Supplementary Table 3.** TFA intake by age-specific distribution among different regions in 1991-2011 (g/d) (Mean±SD). **Supplementary Table 4.** Post hoc tests of TFA intake by age-specific distribution in different regions in each survey year (g/d). **Supplementary Table 5.** TFA intake as the percentage of total energy intake by age-specific distribution among different regions in 1991-2011(*E*%) (Mean±SD). **Supplementary Table 6.** Post hoc tests of TFA intake as the percentage of total energy intake by age-specific distribution in different regions in each survey year (%). **Supplementary Table 7.** Contribution of foods to TFA intake percentage in each survey year by age groups (%).

## Data Availability

All data generated and analyzed in this study are included in this published article and supplementary information files.

## Abbreviations

*E*%: Energy-percent
WHO: World Health Organization
TFAs: Trans fatty acids
CHD: coronary heart disease
CHNS: China Health and Nutrition Survey
CFSA: Chinese Center for Food Safety Risk Assessment
CDC: Chinese Center for Disease Control and Prevention
ANOVA: one-way analysis of variance
