# Supplementary material for: Trans fatty acid intake among Chinese population: a longitudinal study from 1991 to 2011
Source: Lipids Health Dis. 2020 Apr 27;19:80. doi: 10.1186/s12944-020-01247-1 (PMC7184713; doi:10.1186/s12944-020-01247-1)
Supplement: Supplementary file 1 — Additional file 1 : Supplementary Table 1. T test of TFA intake as the percentage of total energy intake by age-specific distribution in specific gender in each survey year (%). Supplementary Table 2. Post hoc test of TFA intake as the percentage of total energy intake in age-specific distribution by gender in each survey year (%). Supplementary Table 3. TFA intake by age-specific distribution among different regions in 1991-2011 (g/d) (Mean±SD). Supplementary Table 4. Post hoc tests of TFA intake by age-specific distribution in different regions in each survey year (g/d). Supplementary Table 5. TFA intake as the percentage of total energy intake by age-specific distribution among different regions in 1991-2011(E%) (Mean±SD). Supplementary Table 6. Post hoc tests of TFA intake as the percentage of total energy intake by age-specific distribution in different regions in each survey year (%). Supplementary Table 7. Contribution of foods to TFA intake percentage in each survey year by age groups (%). [file 12944_2020_1247_MOESM1_ESM.docx]

**Supplementary Table 1.** T test of TFA intake as the percentage of total energy intake by age-specific distribution in specific gender in each survey year (%)

| Year | Age group | *t* | *P^*^* | Post Hoc Test^#^ |
| --- | --- | --- | --- | --- |
| 1991 | ≤18 | -0.705 | 0.480 | A=B |
|  | 19-60 | 2.123 | 0.035 | A>B |
|  | ≥61 | 0.042 | 0.967 | A=B |
| 1993 | ≤18 | -0.413 | 0.679 | A=B |
|  | 19-60 | 2.699 | 0.007 | A>B |
|  | ≥61 | 0.535 | 0.592 | A=B |
| 1997 | ≤18 | -1.615 | 0.106 | A, B |
|  | 19-60 | -0.519 | 0.604 | A=B |
|  | ≥61 | -0.133 | 0.894 | A=B |
| 2000 | ≤18 | -1.715 | 0.087 | A, B |
|  | 19-60 | -0.956 | 0.339 | A=B |
|  | ≥61 | -0.577 | 0.564 | A=B |
| 2004 | ≤18 | -2.171 | 0.030 | A<B |
|  | 19-60 | -2.326 | 0.020 | A<B |
|  | ≥61 | -1.120 | 0.263 | A=B |
| 2006 | ≤18 | 0.640 | 0.523 | A=B |
|  | 19-60 | -1.951 | 0.051 | A, B |
|  | ≥61 | -1.170 | 0.241 | A=B |
| 2009 | ≤18 | -0.905 | 0.365 | A=B |
|  | 19-60 | -2.651 | 0.008 | A<B |
|  | ≥61 | -0.450 | 0.653 | A=B |
| 2011 | ≤18 | -1.433 | 0.152 | A, B |
|  | 19-60 | -2.774 | 0.006 | A<B |
|  | ≥61 | -2.189 | 0.029 | A<B |

^*^When *P*≥0.2 denotes that the mean corresponding to the two samples is equal

^#^A=Males; B=Females

**Supplementary Table 2.** Post hoc test of TFA intake as the percentage of total energy intake in age-specific distribution by gender in each survey year (%)

| Year | Age group | *F* | *P* | Post Hoc Test^*^ |
| --- | --- | --- | --- | --- |
| 1991 | Male | 9.273 | <0.001 | A<B=C |
|  | Female | 5.217 | 0.005 | A=B<C |
| 1993 | Male | 15.49 | <0.001 | A<B=C |
|  | Female | 6.094 | 0.002 | A,B<C |
| 1997 | Male | 2.008 | 0.134 | A=B,B=C |
|  | Female | 1.491 | 0.225 | A=B=C |
| 2000 | Male | 9.106 | <0.001 | A=B<C |
|  | Female | 9.136 | <0.001 | A=B<C |
| 2004 | Male | 3.416 | 0.033 | B=C,C=A |
|  | Female | 7.468 | 0.001 | B=C,A<C |
| 2006 | Male | 1.567 | 0.209 | B=C=A |
|  | Female | 1.235 | 0.291 | A=B=C |
| 2009 | Male | 15.529 | <0.001 | B=C<A |
|  | Female | 13.353 | <0.001 | B=C<A |
| 2011 | Male | 3.252 | <0.039 | C=B, A |
|  | Female | 3.188 | 0.041 | C=B, A |

^*^A: ≤18; B: 19-60; C: ≥60

**Supplementary Table 3.** TFA intake by age-specific distribution among different regions in 1991-2011 (g/d) (Mean±SD)

| Regions | Age group | 1991 | 1993 | 1997 | 2000 | 2004 | 2006 | 2009 | 2011 |
| --- | --- | --- | --- | --- | --- | --- | --- | --- | --- |
| **Northeast** |  |  |  |  |  |  |  |  |  |
| Heilongjiang^*^ | ≤18 |  |  | 0.380(0.020) | 0.382(0.019) | 0.520(0.032) | 0.459(0.020) | 0.389(0.020) | 0.442(0.025) |
|  | 19-60 |  |  | 0.487(0.014) | 0.490(0.013) | 0.517(0.014) | 0.559(0.012) | 0.484(0.009) | 0.581(0.013) |
|  | ≥61 |  |  | 0.480(0.039) | 0.468(0.035) | 0.383(0.027) | 0.482(0.025) | 0.508(0.021) | 0.559(0.025) |
| Liaoning^#^ | ≤18 | 0.211 (0.013) | 0.246(0.013) |  | 0.300(0.017) | 0.458(0.030) | 0.530(0.034) | 0.527(0.038) | 0.447(0.035) |
|  | 19-60 | 0.315(0.012) | 0.353(0.011) |  | 0.367(0.011) | 0.458(0.014) | 0.512(0.015) | 0.486(0.013) | 0.529(0.016) |
|  | ≥61 | 0.284(0.035) | 0.427(0.028) |  | 0.511(0.029) | 0.548(0.029) | 0.553(0.029) | 0.493(0.019) | 0.536(0.026) |
| Central China |  |  |  |  |  |  |  |  |  |
| Henan | ≤18 | 0.192 (0.010) | 0.164(0.009) | 0.244(0.012) | 0.267(0.014) | 0.366(0.018) | 0.327(0.021) | 0.393(0.021) | 0.406(0.023) |
|  | 19-60 | 0.257(0.009) | 0.240(0.008) | 0.331(0.008) | 0.326(0.011) | 0.451(0.013) | 0.478(0.013) | 0.481(0.010) | 0.552(0.018) |
|  | ≥61 | 0.243(0.017) | 0.198(0.015) | 0.319(0.020) | 0.304(0.020) | 0.390(0.019) | 0.411(0.021) | 0.424(0.019) | 0.461(0.021) |
| Hubei**^φ^** | ≤18 | 0.239 (0.009) | 0.239(0.009) | 0.349(0.014) | 0.375(0.018) |  | 0.347(0.025) | 0.443(0.025) | 0.610(0.034) |
|  | 19-60 | 0.353(0.010) | 0.357(0.010) | 0.486(0.011) | 0.475(0.012) |  | 0.504(0.014) | 0.502(0.010) | 0.630(0.014) |
|  | ≥61 | 0.252(0.020) | 0.333(0.023) | 0.348(0.023) | 0.461(0.027) |  | 0.472(0.026) | 0.454(0.017) | 0.602(0.023) |
| Hunan | ≤18 | 0.141 (0.008) | 0.120(0.007) | 0.220(0.013) | 0.200(0.016) | 0.360(0.031) | 0.390(0.027) | 0.353(0.025) | 0.406(0.022) |
|  | 19-60 | 0.196(0.008) | 0.177(0.007) | 0.280(0.009) | 0.300(0.011) | 0.422(0.014) | 0.513(0.015) | 0.408(0.011) | 0.491(0.012) |
|  | ≥61 | 0.206(0.022) | 0.167(0.017) | 0.248(0.020) | 0.275(0.020) | 0.324(0.020) | 0.398(0.025) | 0.356(0.018) | 0.422(0.023) |
| **East Coast** |  |  |  |  |  |  |  |  |  |
| Jiangsu | ≤18 | 0.257 (0.012) | 0.274(0.012) | 0.369(0.017) | 0.477(0.019) | 0.735(0.034) | 0.700(0.034) | 0.737(0.034) | 0.684(0.037) |
|  | 19-60 | 0.359(0.008) | 0.368(0.008) | 0.530(0.013) | 0.562(0.011) | 0.733(0.016) | 0.698(0.015) | 0.690(0.015) | 0.682(0.014) |
|  | ≥61 | 0.343(0.018) | 0.382(0.023) | 0.504(0.022) | 0.532(0.021) | 0.749(0.029) | 0.613(0.021) | 0.603(0.018) | 0.657(0.021) |
| Shandong | ≤18 | 0.218 (0.009) | 0.231(0.011) | 0.385(0.027) | 0.387(0.018) | 0.550(0.032) | 0.436(0.034) | 0.441(0.023) | 0.572(0.035) |
|  | 19-60 | 0.300(0.008) | 0.364(0.010) | 0.430(0.014) | 0.463(0.011) | 0.692(0.016) | 0.593(0.016) | 0.616(0.014) | 0.773(0.017) |
|  | ≥61 | 0.306(0.019) | 0.350(0.019) | 0.401(0.025) | 0.452(0.023) | 0.573(0.025) | 0.573(0.026) | 0.554(0.025) | 0.648(0.026) |
| **Southwest** |  |  |  |  |  |  |  |  |  |
| Guizhou | ≤18 | 0.139(0.009) | 0.129(0.007) | 0.151(0.009) | 0.182(0.011) | 0.215(0.015) | 0.172(0.015) | 0.191(0.015) | 0.276(0.022) |
|  | 19-60 | 0.187(0.008) | 0.188(0.007) | 0.224(0.008) | 0.225(0.009) | 0.327(0.012) | 0.290(0.012) | 0.270(0.010) | 0.367(0.013) |
|  | ≥61 | 0.169(0.016) | 0.194(0.015) | 0.214(0.016) | 0.227(0.018) | 0.243(0.018) | 0.234(0.017) | 0.218(0.020) | 0.311(0.018) |
| Guangxi | ≤18 | 0.210 (0.006) | 0.238(0.008) | 0.331(0.011) | 0.359(0.012) | 0.327(0.014) | 0.326(0.014) | 0.389(0.014) | 0.339(0.012) |
|  | 19-60 | 0.314(0.007) | 0.343(0.008) | 0.419(0.008) | 0.456(0.008) | 0.391(0.008) | 0.453(0.009) | 0.505(0.009) | 0.463(0.007) |
|  | ≥61 | 0.324(0.017) | 0.317(0.013) | 0.372(0.018) | 0.398(0.015) | 0.360(0.018) | 0.417(0.018) | 0.402(0.013) | 0.379(0.012) |

*Heilongjiang province was enrolled as the ninth province in 1997.

^#^The data of Liaoning province was unavailable in 1997 and it rejoined in 2000.

**^φ^**Hubei province did not conduct an investigation into cooking oil in 2004 and its TFA intake was unavailable.

**Supplementary Table 4.** Post hoc tests of TFA intake by age-specific distribution in different regions in each survey year (g/d)

| Year | Age group | F | *P* | Post Hoc Tests^*^ |
| --- | --- | --- | --- | --- |
| 1991 | ≤18 | 21.392 | <0.001 | I=G<E=H=A=D, H=A=D=F, A=D=F=C |
|  | 19-60 | 57.371 | <0.001 | I=G<E<D=H=A, A=F, F=C |
|  | ≥61 | 10.017 | <0.001 | I=G, G=E=F=A, E=F=A=D, A=D=H=C |
| 1993 | ≤18 | 40.269 | <0.001 | G=I<E<D=H=F=A=C |
|  | 19-60 | 92.649 | <0.001 | G=I<E<H=A=F=D=C |
|  | ≥61 | 24.254 | <0.001 | G=I=E<H=F=D=C, F=D=C=A |
| 1997 | ≤18 | 33.572 | <0.001 | I<G=E<H=F=C=B=D |
|  | 19-60 | 99.808 | <0.001 | I<G<E<H=D<F=B=C |
|  | ≥61 | 20.157 | <0.001 | I=G, G=E, E=F=H=D, D=B, B=C |
| 2000 | ≤18 | 36.240 | <0.001 | I=G<E=A, A=H, H=F=B=D<C |
|  | 19-60 | 100.05 | <0.001 | I<G=E, E=A<H=D=F=B<C |
|  | ≥61 | 24.820 | <0.001 | I=G=E<H=D=F=B, D=F=B=A=C |
| 2004 | ≤18 | 66.455 | <0.001 | I=H, H=G=E, G=E=A, A=B=D<C |
|  | 19-60 | 227.720 | <0.001 | I=H, H=G, G=E=A, A=B<D=C |
|  | ≥61 | 78.679 | <0.001 | I<G=H=B=E<A=D<C |
| 2006 | ≤18 | 42.453 | <0.001 | I<H=E=F=G, F=G=D=B, D=B=A<C |
|  | 19-60 | 62.699 | <0.001 | I<H=E, E=F=A=G, F=A=G=B, B=D<C |
|  | ≥61 | 28.661 | <0.001 | I<G=E=H=F=B, F=B=A, B=A=D, A=D=C |
| 2009 | ≤18 | 42.970 | <0.001 | I<G=B=H=E=D=F, D=F=A<C |
|  | 19-60 | 98.018 | <0.001 | I<G<E=B=A=F=H<D<C |
|  | ≥61 | 41.314 | <0.001 | I<G=H=E, H=E=F, E=F=A, F=A=B, A=B=D, B=D=C |
| 2011 | ≤18 | 29.094 | <0.001 | I=H, H=E=G, E=G=B=A, A=D, D=F=C |
|  | 19-60 | 72.791 | <0.001 | I<H=G, G=A, A=E=B, B=F, F=C<D |
|  | ≥61 | 35.362 | <0.001 | I<H=G, G=E, E=A=B, A=B=F, B=F=D=C |

^*^A=Liaoning, B=Heilongjiang, C=Jiangsu, D=Shandong, E=Henan, F=Hubei, G=Hunan, H=Guangxi, I=Guizhou

**Supplementary Table 5.** TFA intake as the percentage of total energy intake by age-specific distribution among different regions in 1991-2011(*E*%) (Mean±SD)

| Regions | Age group | 1991 | 1993 | 1997 | 2000 | 2004 | 2006 | 2009 | 2011 |
| --- | --- | --- | --- | --- | --- | --- | --- | --- | --- |
| **Northeast** |  |  |  |  |  |  |  |  |  |
| Heilongjiang* | ≤18 |  |  | 0.182(0.008) | 0.210(0.010) | 0.250(0.010) | 0.238(0.009) | 0.220(0.012) | 0.244(0.013) |
|  | 19-60 |  |  | 0.169(0.004) | 0.199(0.004) | 0.207(0.004) | 0.226(0.004) | 0.208(0.003) | 0.254(0.005) |
|  | ≥61 |  |  | 0.184(0.013) | 0.200(0.011) | 0.174(0.009) | 0.218(0.009) | 0.228(0.008) | 0.263(0.009) |
| Liaoning^#^ | ≤18 | 0.105 (0.007) | 0.124(0.006) |  | 0.144(0.007) | 0.224(0.012) | 0.259(0.015) | 0.289(0.018) | 0.276(0.016) |
|  | 19-60 | 0.104(0.004) | 0.135(0.004) |  | 0.141(0.004) | 0.190(0.005) | 0.192(0.005) | 0.203(0.004) | 0.243(0.006) |
|  | ≥61 | 0.103(0.012) | 0.160(0.011) |  | 0.218(0.011) | 0.231(0.010) | 0.230(0.011) | 0.232(0.008) | 0.260(0.010) |
| Central China |  |  |  |  |  |  |  |  |  |
| Henan | ≤18 | 0.107 (0.006) | 0.102(0.007) | 0.120(0.005) | 0.129(0.005) | 0.196(0.009) | 0.190(0.010) | 0.223(0.008) | 0.264(0.012) |
|  | 19-60 | 0.121(0.005) | 0.110(0.004) | 0.116(0.003) | 0.124(0.003) | 0.169(0.004) | 0.187(0.004) | 0.195(0.004) | 0.234(0.005) |
|  | ≥61 | 0.130(0.013) | 0.098(0.009) | 0.134(0.007) | 0.138(0.007) | 0.180(0.007) | 0.197(0.010) | 0.191(0.007) | 0.219(0.007) |
| Hubei**^φ^** | ≤18 | 0.121(0.004) | 0.134(0.005) | 0.173(0.005) | 0.164(0.006) |  | 0.167(0.009) | 0.242(0.011) | 0.278(0.010) |
|  | 19-60 | 0.143(0.004) | 0.147(0.004) | 0.176(0.004) | 0.166(0.003) |  | 0.175(0.004) | 0.191(0.004) | 0.237(0.004) |
|  | ≥61 | 0.108(0.008) | 0.148(0.009) | 0.158(0.009) | 0.180(0.008) |  | 0.183(0.008) | 0.204(0.007) | 0.245(0.006) |
| Hunan | ≤18 | 0.067 (0.003) | 0.062(0.004) | 0.087(0.004) | 0.082(0.007) | 0.137(0.009) | 0.171(0.010) | 0.182(0.011) | 0.227(0.010) |
|  | 19-60 | 0.069(0.003) | 0.075(0.003) | 0.094(0.003) | 0.099(0.003) | 0.140(0.004) | 0.184(0.005) | 0.162(0.004) | 0.200(0.004) |
|  | ≥61 | 0.076(0.007) | 0.071(0.007) | 0.101(0.008) | 0.104(0.006) | 0.122(0.007) | 0.162(0.008) | 0.157(0.008) | 0.189(0.008) |
| **East Coast** |  |  |  |  |  |  |  |  |  |
| Jiangsu | ≤18 | 0.121 (0.005) | 0.139(0.005) | 0.189(0.007) | 0.218(0.009) | 0.331(0.013) | 0.340(0.013) | 0.360(0.015) | 0.359(0.014) |
|  | 19-60 | 0.132(0.003) | 0.144(0.003) | 0.194(0.004) | 0.194(0.003) | 0.248(0.004) | 0.247(0.005) | 0.249(0.004) | 0.277(0.005) |
|  | ≥61 | 0.139(0.007) | 0.158(0.009) | 0.216(0.008) | 0.206(0.007) | 0.269(0.008) | 0.253(0.008) | 0.245(0.007) | 0.286(0.007) |
| Shandong | ≤18 | 0.108 (0.005) | 0.115(0.005) | 0.176(0.009) | 0.182(0.007) | 0.263(0.013) | 0.252(0.019) | 0.272(0.014) | 0.318(0.013) |
|  | 19-60 | 0.122(0.004) | 0.141(0.004) | 0.160(0.004) | 0.182(0.004) | 0.260(0.005) | 0.223(0.005) | 0.246(0.005) | 0.308(0.005) |
|  | ≥61 | 0.132(0.008) | 0.150(0.007) | 0.171(0.009) | 0.214(0.008) | 0.266(0.010) | 0.254(0.009) | 0.271(0.009) | 0.304(0.008) |
| **Southwest** |  |  |  |  |  |  |  |  |  |
| Guizhou | ≤18 | 0.063(0.004) | 0.062(0.003) | 0.070(0.004) | 0.092(0.005) | 0.092(0.006) | 0.083(0.006) | 0.101(0.007) | 0.158(0.009) |
|  | 19-60 | 0.061(0.002) | 0.063(0.002) | 0.071(0.002) | 0.082(0.003) | 0.106(0.004) | 0.098(0.004) | 0.106(0.004) | 0.155(0.005) |
|  | ≥61 | 0.067(0.006) | 0.074(0.005) | 0.085(0.006) | 0.099(0.007) | 0.093(0.006) | 0.094(0.006) | 0.090(0.006) | 0.146(0.006) |
| Guangxi | ≤18 | 0.144 (0.004) | 0.143(0.004) | 0.155(0.004) | 0.172(0.005) | 0.167(0.005) | 0.182(0.006) | 0.226(0.006) | 0.221(0.007) |
|  | 19-60 | 0.141(0.003) | 0.142(0.003) | 0.155(0.003) | 0.169(0.003) | 0.154(0.003) | 0.188(0.003) | 0.210(0.003) | 0.201(0.003) |
|  | ≥61 | 0.170(0.007) | 0.156(0.005) | 0.159(0.005) | 0.180(0.005) | 0.160(0.005) | 0.196(0.006) | 0.197(0.005) | 0.192(0.005) |

*Heilongjiang province was enrolled as the ninth province in 1997.

^#^The data of Liaoning province was unavailable in 1997 and it rejoined in 2000.

**^φ^**Hubei province did not conduct an investigation into cooking oil in 2004 and its TFA intake was unavailable.

**Supplementary Table 6.** Post hoc tests of TFA intake as the percentage of total energy intake by age-specific distribution in different regions in each survey year (%)

| Year | Age group | *F* | *P* | Post Hoc Tests^*^ |
| --- | --- | --- | --- | --- |
| 1991 | ≤18 | 39.407 | <0.001 | I=G<A=E=D=C=F<H |
|  | 19-60 | 89.781 | <0.001 | I=G<A<E=D<C<H=F |
|  | ≥61 | 18.512 | <0.001 | I=G, A=F=E=D=C<H |
| 1993 | ≤18 | 47.814 | <0.001 | G=I<E=D=A, A=F=C=H |
|  | 19-60 | 97.675 | <0.001 | I=G<E<D=A=F=C=H |
|  | ≥61 | 26.992 | <0.001 | G=I=E<F=D=H=C=A |
| 1997 | ≤18 | 62.737 | <0.001 | I=G<E<H, F=D=B=C |
|  | 19-60 | 172.87 | <0.001 | I<G<E<H=D=B, B=F<C |
|  | ≥61 | 29.938 | <0.001 | I=G<E=F, F=H=D=B, B=C |
| 2000 | ≤18 | 49.567 | <0.001 | G=I<E=A, A=F=H, F=H=D, D=B, B=C |
|  | 19-60 | 147.09 | <0.001 | G<I<E<A<F=H, F<D=C, C=B |
|  | ≥61 | 38.198 | <0.001 | I=G<E<F=H=B=C, B=C=D=A |
| 2004 | ≤18 | 89.205 | <0.001 | I=G, G=H, H=E, E=A, A=B=D<C |
|  | 19-60 | 318.89 | <0.001 | I<G=H, H=E, A=B<C=D |
|  | ≥61 | 106.91 | <0.001 | I=G<H=B=E<A=D, D=C |
| 2006 | ≤18 | 56.998 | <0.001 | I<F=G=H=E<B=D=A<C |
|  | 19-60 | 92.406 | <0.001 | I<F=G=E=H=A<D=B, D<C |
|  | ≥61 | 39.031 | <0.001 | I<G=F=H=E, H=E=B=A, B=A=C=D |
| 2009 | ≤18 | 51.665 | <0.001 | I<G=B, B=E=H=F, F=D=A<C |
|  | 19-60 | 113.73 | <0.001 | I<G<F=E=A, E=A=B, A=B=H<D=C |
|  | ≥61 | 61.685 | <0.001 | I<G<E=H=F=B, F=B=A, B=A=C, C=D |
| 2011 | ≤18 | 39.407 | <0.001 | I<H=G=B, G=B=E=A, B=E=A=F, A=F=D, D=C |
|  | 19-60 | 92.223 | <0.001 | I<G=H<E=F=A=B<C<D |
|  | ≥61 | 51.111 | <0.001 | I<G=H=E, E=F, F=A=B, A=B=C, C=D |

^*^A=Liaoning, B=Heilongjiang, C=Jiangsu, D=Shandong, E=Henan, F=Hubei, G=Hunan, H=Guangxi, I=Guizhou

**Supplementary Table 7.** Contribution of foods to TFA intake percentage in each survey year by age groups (%)

|  | | 1991 | 1993 | 1997 | 2000 | 2004 | 2006 | 2009 | 2011 |
| --- | --- | --- | --- | --- | --- | --- | --- | --- | --- |
| ≤18 | **Natural TFA intake** |  |  |  |  |  |  |  |  |
|  | Pork products | 7.39 | 8.12 | 5.86 | 6.3 | 5.39 | 5.58 | 5.66 | 4.13 |
|  | Mutton & beef | 4.01 | 6.75 | 4.7 | 5.53 | 5.87 | 4.39 | 3.68 | 3.73 |
|  | Poultry & poultry products | 3.66 | 4.27 | 4.87 | 4.58 | 4.88 | 4.57 | 5.45 | 5.42 |
|  | Milk & milk products | 3.14 | 2.96 | 1.95 | 4.76 | 12.07 | 11.32 | 11.97 | 17.25 |
|  | Total | 18.20 | 22.10 | 17.38 | 21.17 | 28.21 | 25.86 | 26.76 | 30.53 |
|  | **Industrial TFA intake** |  |  |  |  |  |  |  |  |
|  | Butter & cream | 0.02 | 0.12 | 0.04 | 0.08 | 0.15 | 0.08 | 0.11 | 0.05 |
|  | Ethnic foods | 3.40 | 4.34 | 4.60 | 1.92 | 3.36 | 3.62 | 3.97 | 3.13 |
|  | Bakery product | 5.75 | 3.20 | 4.32 | 3.84 | 6.25 | 7.29 | 11.37 | 14.45 |
|  | Fast foods | 0 | 0 | 0.69 | 0.91 | 1.59 | 3.09 | 3.59 | 4.01 |
|  | Solid beverage | 0.02 | 0.01 | 0.08 | 0.05 | 0 | 0 | 0.08 | 0.11 |
|  | Ice creams | 0.12 | 0.08 | 0.03 | 0.09 | 0.05 | 0.06 | 0.32 | 0.19 |
|  | Chocolate candy | 0.23 | 0.14 | 0.22 | 0.16 | 0.06 | 0.02 | 0.83 | 1.38 |
|  | Vegetable oil | 64.60 | 61.07 | 64.60 | 66.05 | 54.54 | 53.66 | 47.75 | 42.45 |
|  | Condiments (Sauces & oils) | 3.71 | 2.89 | 4.39 | 2.14 | 0.68 | 1.02 | 0.81 | 0.78 |
|  | Puffed food | 0.34 | 0.11 | 0.09 | 0.21 | 0.05 | 0.19 | 0.25 | 0.40 |
|  | Youbing & youtiao | 3.61 | 5.94 | 3.56 | 3.39 | 5.06 | 5.11 | 4.17 | 2.53 |
|  | Total | 81.80 | 77.90 | 82.62 | 78.84 | 71.79 | 74.14 | 73.25 | 69.48 |
| 19-60 | **Natural TFA intake** |  |  |  |  |  |  |  |  |
|  | Pork products | 7.50 | 7.74 | 5.59 | 6.15 | 5.43 | 5.39 | 5.72 | 4.66 |
|  | Mutton & beef | 4.79 | 7.17 | 5.72 | 5.62 | 6.22 | 5.37 | 4.55 | 4.06 |
|  | Poultry & poultry products | 4.08 | 4.42 | 4.75 | 4.82 | 4.75 | 4.31 | 5.73 | 5.27 |
|  | Milk & milk products | 1.47 | 1.61 | 0.72 | 2.13 | 4.75 | 3.69 | 3.57 | 3.06 |
|  | Total | 17.84 | 20.94 | 16.78 | 18.72 | 21.15 | 18.76 | 19.57 | 17.05 |
|  | **Industrial TFA intake** |  |  |  |  |  |  |  |  |
|  | Butter & cream | 0 | 0.18 | 0.01 | 0.12 | 0.04 | 0.05 | 0.02 | 0 |
|  | Ethnic foods | 3.03 | 4.06 | 3.85 | 1.97 | 5.12 | 5.91 | 6.15 | 7.12 |
|  | Bakery product | 2.27 | 1.03 | 1.78 | 2.04 | 2.84 | 3.90 | 4.91 | 5.94 |
|  | Fast foods | 0 | 0 | 0.22 | 0.24 | 0.58 | 1.60 | 2.27 | 2.63 |
|  | Solid beverage | 0.02 | 0.02 | 0.01 | 0.01 | 0.06 | 0.01 | 0.02 | 0.11 |
|  | Ice creams | 0.01 | 0.05 | 0 | 0 | 0.01 | 0.02 | 0.03 | 0.03 |
|  | Chocolate candy | 0.03 | 0.04 | 0.22 | 0.06 | 0.03 | 0.08 | 0.04 | 0.11 |
|  | Vegetable oil | 69.79 | 64.35 | 69.74 | 70.75 | 63.54 | 61.77 | 59.29 | 59.29 |
|  | Condiments (Sauces & oils) | 3.35 | 2.69 | 4.18 | 2.08 | 0.71 | 0.88 | 0.98 | 0.68 |
|  | Puffed food | 0.22 | 0.16 | 0.04 | 0.03 | 0.07 | 0.48 | 0.83 | 1.05 |
|  | Youbing & youtiao | 3.43 | 6.49 | 3.16 | 3.97 | 5.84 | 6.55 | 5.90 | 6.00 |
|  | Total | 82.15 | 79.07 | 83.21 | 81.27 | 78.84 | 81.25 | 80.44 | 82.96 |
| ≥61 | **Natural TFA intake** |  |  |  |  |  |  |  |  |
|  | Pork products | 7.25 | 7.51 | 5.90 | 5.79 | 5.01 | 4.97 | 5.41 | 4.12 |
|  | Mutton & beef | 2.96 | 6.30 | 4.73 | 4.87 | 3.83 | 3.05 | 2.69 | 2.52 |
|  | Poultry & poultry products | 3.42 | 4.37 | 3.81 | 4.32 | 3.81 | 3.36 | 4.34 | 4.03 |
|  | Milk & milk products | 3.43 | 2.33 | 2.21 | 5.17 | 7.98 | 7.04 | 6.55 | 5.94 |
|  | Total | 17.06 | 20.51 | 16.65 | 20.15 | 20.63 | 18.42 | 18.99 | 16.61 |
|  | **Industrial TFA intake** |  |  |  |  |  |  |  |  |
|  | Butter & cream | 0 | 0 | 0 | 0 | 0 | 0.12 | 0 | 0 |
|  | Ethnic foods | 3.17 | 3.56 | 3.60 | 1.91 | 5.33 | 4.93 | 4.87 | 5.98 |
|  | Bakery product | 3.28 | 1.44 | 2.56 | 2.89 | 4.05 | 4.80 | 6.36 | 6.48 |
|  | Fast foods | 0 | 0 | 0.17 | 0.24 | 0.44 | 1.21 | 1.79 | 1.72 |
|  | Solid beverage | 0.03 | 0.02 | 0.04 | 0 | 0.04 | 0 | 0.02 | 0.01 |
|  | Ice creams | 0.08 | 0 | 0 | 0 | 0 | 0.01 | 0 | 0.02 |
|  | Chocolate candy | 0 | 0 | 0.23 | 0.04 | 0.05 | 0.02 | 0.01 | 0.07 |
|  | Vegetable oil | 71.02 | 66.16 | 69.90 | 69.17 | 63.85 | 62.79 | 61.25 | 63.22 |
|  | Condiments (Sauces & oils) | 2.73 | 2.28 | 4.11 | 1.93 | 0.65 | 1.14 | 0.68 | 0.73 |
|  | Puffed food | 0 | 0.20 | 0.03 | 0.05 | 0.18 | 0.53 | 1.15 | 0.95 |
|  | Youbing & youtiao | 2.63 | 5.83 | 2.71 | 3.62 | 4.79 | 6.02 | 4.89 | 4.22 |
|  | Total | 82.94 | 79.49 | 83.65 | 79.85 | 79.38 | 81.57 | 81.02 | 83.40 |
